# Myo-inositol supplementation improves cardiometabolic factors, anthropometric measures, and liver function in obese patients with non-alcoholic fatty liver disease

**DOI:** 10.3389/fnut.2023.1092544

**Published:** 2023-02-07

**Authors:** Sara Arefhosseini, Neda Roshanravan, Helda Tutunchi, Somayyeh Rostami, Manuchehr Khoshbaten, Mehrangiz Ebrahimi-Mameghani

**Affiliations:** ^1^Student Research Committee, Tabriz University of Medical Sciences, Tabriz, Iran; ^2^Cardiovascular Research Center, Tabriz University of Medical Sciences, Tabriz, Iran; ^3^Endocrine Research Center, Tabriz University of Medical Sciences, Tabriz, Iran; ^4^Department of Internal Medicine, Faculty of Medicine, Tabriz University of Medical Sciences, Tabriz, Iran; ^5^Nutrition Research Center, Department of Biochemistry and Diet Therapy, Faculty of Nutrition and Food Sciences, Tabriz University of Medical Sciences, Tabriz, Iran

**Keywords:** anthropometric measures, glycemic indices, lipid profile, myo-inositol, non-alcoholic fatty liver disease, NAFLD, obesity

## Abstract

**Background:**

Non-alcoholic fatty liver disease (NAFLD) as the hepatic manifestation of metabolic syndrome is closely associated with type 2 diabetes mellitus. Myo-inositol (MI)—a 6-C sugar alcohol—with insulin-mimetic, anti-diabetic, lipid-lowering, and anti-inflammatory properties has exerted favorable effects on insulin resistance-related disorders and metabolic disease, while recent animal studies revealed its positive effects on liver function. This study aimed to investigate the effects of MI supplementation on cardiometabolic factors, anthropometric measures, and liver function in obese patients with NAFLD.

**Methods:**

This double-blinded placebo-controlled randomized clinical trial was carried out on 48 obese patients with NAFLD who were randomly assigned to either MI (4g/day) or placebo (maltodextrin 4g/day) along with dietary recommendations for 8 weeks. Glycemic indices, lipid profile, liver enzymes anthropometric measures, and blood pressure were evaluated pre- and post-intervention. Dietary intakes were assessed using a 3-day 24 h recall and analyzed by Nutritionist IV software. Insulin resistance was estimated using the homeostasis model assessment of insulin resistance (HOMA-IR), and beta-cell function (HOMA-B) was also estimated.

**Results:**

Anthropometric measures decreased significantly in both groups, while the reduction in weight (*p* = 0.049) and systolic blood pressure (*p* = 0.006) in the MI group was significantly greater than in the placebo group after adjusting for baseline values and energy intake. Although energy and macronutrient intakes decreased significantly in both groups, between-group differences were not significant after adjusting for the potential confounders. MI supplementation led to a significant reduction in serum fasting insulin (*p* = 0.008) and HOMA-IR (*p* = 0.046). There were significant improvements in lipid profile, liver enzymes, and aspartate aminotransferase/alanine aminotransferase ratio as well as serum ferritin level in the MI group, compared to the placebo group at the endpoint. By MI supplementation for eight weeks, 1 in 3 patients reduced one- grade in the severity of NAFLD.

**Conclusion:**

MI supplementation could significantly improve IR, lipid profile, and liver function in patients with NAFLD. Further clinical trials with larger sample sizes, longer duration, different MI doses, and other inositol derivatives are recommended.

## Introduction

Over the last decades, the most common etiology of chronic liver diseases has been non-alcoholic fatty liver disease (NAFLD) (1). Various estimates of NAFLD prevalence have been reported in the general population. By 2030, the prevalence of NAFLD among the adult population is projected at 33.5% (2). NAFLD—as an umbrella term—refers to different types of fatty liver diseases, ranging from simple steatosis to hepatic fibrosis in the absence of alcohol consumption (3). “Multi-hit” pathogenesis has been introduced for NAFLD, characterized by the synergic role of genetic and epigenetic factors, including insulin resistance (IR), inflammation, oxidative stress, and changes in gut microbiota (4). NAFLD is closely associated with type 2 diabetes mellitus (T2DM) in a bi-directional way (5). It is estimated that about three out of four patients with diabetes suffer from NAFLD (6). IR, as the fundamental core in both of these conditions, is defined by disturbances in intra-cellular insulin signaling pathways such as phosphatidylinositide 3-kinase/Akt (PI3K/AKT) pathway (7). Furthermore, simple steatosis at the beginning of NAFLD is followed by a number of metabolic abnormalities such as reduced hepatic fatty acid oxidation and enhanced *de novo* lipogenesis and adipose tissue lipolysis that eventually lead to IR (7, 8). In turn, IR is linked to other endocrine and metabolic disorders such as obesity, polycystic ovary syndrome (PCOs), metabolic syndrome (Mets), and cardiovascular diseases (CVD) (7). Recent evidence indicates the inter-relationship between obesity and NAFLD, proposing the role of adipose tissue in regulating endocrine signaling pathways such as hormones, adipokines, and pro-inflammatory cytokines (9, 10). Accumulating evidence suggests that NAFLD is the hepatic manifestation of Mets due to the coexistence of visceral obesity, IR, dyslipidemia, and hypertension. Recently, it has been recommended to rename NAFLD as metabolic dysfunction-associated fatty liver disease (MAFLD) (4, 11).

Currently, there is not a confirmed therapeutical approach; however, conjugated therapy targeting improvements in lifestyle (such as weight reduction and dietary modification) along with supplementation/medication (e.g., glucose and lipid-lowering agents) have been frequently applied in the management of NAFLD (12). Inositols (INS), as 6-C sugar alcohol derivatives, are natural supplements that have exhibited a plethora of pharmacological properties in the management of IR-related conditions (13). Myo-inositol (MI)—a cyclic carbohydrate with six hydroxyl groups—is the predominant isoform of INS and is endogenously formed from D-glucose by the kidneys and the liver in the human body up to 4 g/day (14). Beans, nuts, seeds, grains, vegetables, and fruits are among the main dietary sources of MI (14, 15). MI mediates a large number of eukaryotic cellular processes, including cell growth and survival, ATP production, energy homeostasis, osmoregulation, and insulin-mimetic feature *via* acting as a secondary messenger or neurotransmitter (in the form of INS glycans and INS triphosphate) (16).

In addition, MI has shown a wide range of therapeutical aspects, e.g., anti-inflammatory/antioxidant, anti-cancer, and, particularly, anti-diabetic properties (16). A large number of clinical trials have revealed the favorable effects of MI on the management of IR-related diseases such as T2DM, PCOs, and Mets (17). The insulin-mimetic property of MI enhances insulin sensitivity accompanied by alleviating metabolic disturbances, gene expressions, inflammatory pathways, oxidative stress biomarkers, and hormonal states (16, 18). Therefore, it appears that MI could be a powerful ingredient in the management of NAFLD as well. Recent animal studies have reported positive effects of MI on liver steatosis, oxidative stress, and inflammation (19). To the best of our knowledge, there is only one human clinical trial assessing the effect of INS supplementation (in the form of D-pinitol) on patients with NAFLD (20). Accordingly, the present randomized clinical trial (RCT) aimed to investigate the effects of MI supplementation on cardiometabolic factors, anthropometric measures, and liver function in obese patients with NAFLD.

## Materials and methods

### Study design

To investigate the effects of MI on cardiometabolic factors and liver function, this double-blind placebo-controlled RCT was conducted on patients with NAFLD. This trial design was approved by the Ethics Committee of Research vice-chancellor of Tabriz University of Medical Sciences, Tabriz, Iran (Ethics code: TBZMED. REC.1400.567), and also registered in the Iranian Registry of Clinical Trials (IRCT20100209003320N22). An informed consent form was read and signed by the patients at the beginning of the trial.

### Participants

Fifty-one obese men and women (Body mass index (BMI) = 30–40 Kg/m^2^) aged 18–55 years with mild and moderate NAFLD referred from specialized and sub-specialized clinics of Tabriz University of Medical Sciences, Tabriz, Iran, were recruited and confirmed by a gastroenterologist. NAFLD diagnosis was performed by a radiologist, using ultrasonography (Sonoace X4 Medisio, South Korea) in a fasting state. The severity of liver steatosis was assessed by an ultrasonographist and classified into three grades, i.e., grade I as “*mild,”* grade II as “*moderate”* based on Hamaguchi et al. (21). Those who were pregnant, lactating, menopause, alcohol drinker, smoker, following special diets or taking any herbal and dietary supplements over the past 3 months, taking medications affecting lipid and glucose metabolism (e.g., glucose and lipid-lowering or anti-hypertensive medications), contraceptives and those suffering from liver, kidney, and gastro-intestinal diseases as well as metabolic disorders (e.g., T2DM, PCOs, and cancer) were excluded.

### Sample size

Changes in serum triglyceride (TG) levels by Lee et al. (20) among patients with NAFLD who consumed D-pinitol were applied for sample size estimation, using power analysis and sample size software (PASS; NCSS, LLC, US). By considering 95% confidence interval (CI) and 80% power, the obtained sample size for each group was 18. Moreover, we increased sample size to 24 persons in each group by supposing a 30% drop-out rate.

### Randomization, blinding, and intervention

Random Allocation Software (RAS) and randomized block procedure were applied for allocating the patients with NAFLD into one of the two experimental groups (i.e., MI and placebo) (1:1) by a research assistant who was not involved in the study and randomized block procedure of size 3 ]gender (female vs. male), age (18–35 yrs. vs. 36–55 yrs.), and BMI (< 35 kg/m^2^ vs. ≥35 kg/m^2^)]. The research assistant packaged and prepared the supplement and placebo sachets with a three-digit code for each of the treatments. The patients, assessors, and researchers were blinded to the study allocation. The assignment was concealed from the researcher before randomization for treatment.

MI powder (Wholesale Health Connection, China) was packaged into 2 g sachets in hygienic condition. The patients in the MI group (*N* = 25) received MI sachets (2 g) twice a day by dissolving in one glass of water before lunch and dinner, while those in the placebo group (*N* = 26) received maltodextrin sachets (2 g) twice a day by dissolving in one glass of water before lunch and dinner for 8 weeks. The MI and maltodextrin sachets were completely similar and identical in size, color, and all other aspects. The sachets were delivered every 2 weeks, and the patients were asked to return unused sachets fortnightly to assess the compliance rate. Healthy dietary recommendations for weight loss were given to all patients, and changes in weight were assessed every 2 weeks (22). The patients were also asked to maintain their usual lifestyle habits and follow the dietary recommendations.

### Assessment of anthropometric measures, physical activity, dietary intake, and blood pressure

At baseline, demographic and disease details were collected for each patient. Anthropometric measurements, physical activity levels, and dietary intakes were assessed at the beginning and end of the trial.

Weight and stature were measured with minimal clothing and without shoes using a Seca stadiometer (Hamburg, Germany) to the nearest 100 g and 0.5 cm, respectively. Then, BMI was estimated as weight (Kg) divided by height squared (m^2^). The circumferences of the neck (NC) and waist (WC) were also assessed using non-stretchable tape halfway between the base of the neck and the upper part of the sternum and halfway between the lower ribs and the iliac crest to the nearest 0.1 cm, respectively. The ratios of NC to height and WC were also estimated.

The International Physical Activity Questionnaire-Short Form (IPAQ-SF) was applied for assessing physical activity levels through a face-to-face interview. The patients reported how much time they spent doing each of the defined intensity-varied activities during the past week. The metabolic equivalent of task (MET-hours/week) score was calculated to classify the patients into “high,” “moderate,” or “low” levels of activity based on the manual (23).

A 3-day food recall (2 weekdays and a weekend) was used to assess dietary intakes at baseline and end of the trial. Daily dietary data were analyzed using Nutritionist IV software modified for Iranian foods (First Databank, San Bruno, CA, USA) to obtain energy and macronutrients intakes.

Systolic (SBP) and diastolic blood pressure (DBP) were assessed after 15 min resting in a seated position using an automated digital sphygmomanometer (Microlife A100–30, Berneck, Switzerland). The measurement was repeated three times with a 5-min interval, and the mean of three measurements was used for data analysis.

### Laboratory assays

Venus blood samples were collected after 12–14 h overnight fasting from each patient. Serum was separated and stored at −80°C until assays. Fasting serum was used to assess fasting blood sugar (FBS), total cholesterol (TC), high-density lipoprotein cholesterol (HDL-c), and TG based on colorimetric-enzymatic methods using commercial kits (Pars-Azmoon Co., Tehran, Iran), and then, low-density lipoprotein cholesterol (LDL-c) was estimated using Friedewald equation (24). Serum alanine aminotransferase (ALT) and aspartate transaminase (AST) concentrations were assessed at baseline and at the end of the study using the International Federation of Clinical Chemistry (IFCC) approved method (25). Hemoglobin A1C (HbA1c) was assessed using photometry in whole blood using a Pars Azmoun Company kit (Pars Azmoun, Iran) and Hitachi auto-analyzer (Hitachi-917, Tokyo, Japan). Insulin and ferritin were quantified using the enzyme-linked immunosorbent assay (ELISA) method and commercial kits (Monobind, Lake Forest, CA, USA). For assessing IR, the homeostatic model assessment for IR (HOMA-IR) and homeostasis beta-cell function (HOMA-B) were applied as follows (26):

HOMA-IR = [fasting insulin (μIU/mL) × fasting glucose (mg/dL)]/405.

HOMA-B = 360 × fasting insulin (μU/mL)/(fasting glucose (mg/dL) – 63).

### Study outcomes

Changes in serum glycemic indices, lipid profiles, blood pressure, energy and macronutrient intakes, and anthropometric indices were considered the primary outcomes of this trial, while changes in the serum levels of liver enzymes and NAFLD grade were the secondary outcomes.

### Statistical analysis

SPSS Statistics software (IBM SPSS Statistics, Armonk, USA, latest version) was applied for data entry and statistical analysis. The Kolmogorov–Smirnov test was used for assessing the distribution of continuous variables and expressed by mean ± standard deviation (SD) whereas frequency (percentage) for categorical variables. After the treatment approach was used for both primary and secondary outcomes, between-group and within-group differences were tested using independent samples *t*-test and paired samples *t*-test for continuous variables, respectively. Intra- and inter-group differences of the qualitative variables were done using the sign and chi-square tests, respectively. At the end of the trial, inter-group changes were examined using the analysis of covariance (ANCOVA) test by adjusting for the confounders (i.e., baseline values and energy intake). To assess the effectiveness of treatment with medication, the number needed to treat (NNT) was calculated for one- and two-grade reduction in liver steatosis based on the following formula: NNT = 1/Absolute risk reduction (ARR). The significance level was defined at a *p-*value lower than 0.05.

## Results

**Figure 1:**
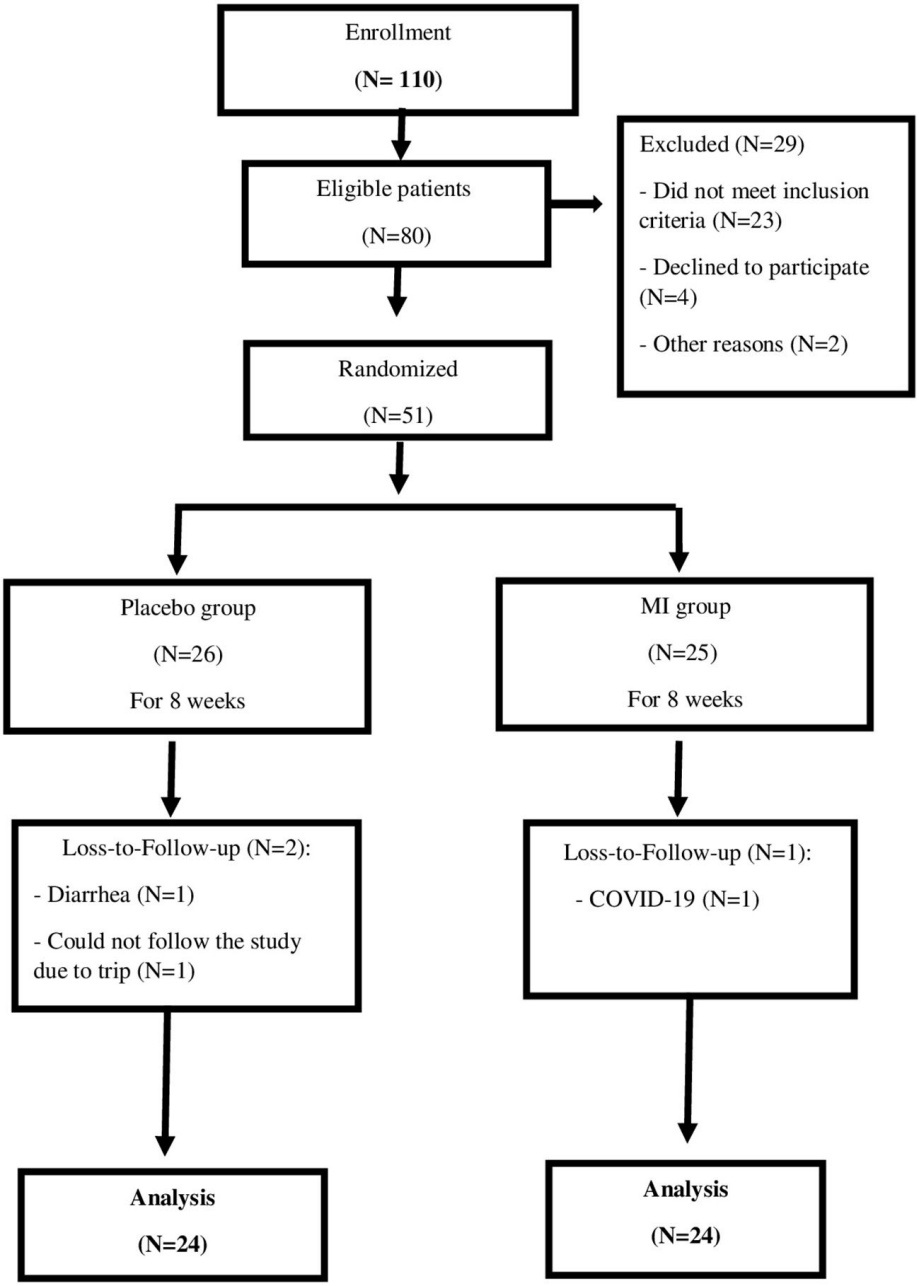
Study flow chart.

Of 51 patients, 48 subjects (24 patients in each group) completed the trial while two patients in the placebo group and one patient in the MI group dropped out for reasons unrelated to the interventions (Figure 1).

Table 1 demonstrates baseline characteristics in the two studied groups. Approximately half of the studied patients in both groups were women, and most of them were married. At baseline, there were no statistically significant differences in baseline characteristics between the two groups.

**Table 1:** Baseline characteristics of the study participants.

**Variable**	**Placebo** **(*N =* 24)** **Mean ±SD**	**MI** **(*N =* 24)** **Mean ±SD**	***p***
Age (yr.)	37.50 ± 6.86	36.33 ± 8.49	0.603^*^
Weight (Kg)	96.93 ± 14.78	96.43 ± 14.68	0.907^*^
Height (m)	1.68 ± 0.10	1.69 ± 0.10	0.859^*^
NC (cm)	40.15 ± 3.76	39.54 ± 3.50	0.565^*^
WC (cm)	111.33 ± 9.21	109.85 ± 9.57	0.588^*^
BMI (Kg/m^2^)	34.16 ± 3.38	33.77 ± 3.53	0.701^*^
PAL (MET-min/wk)	57.54 ± 39.42	63.53 ± 37.17	0.590^*^
	%	%	
Female	52.4	45.8	0.594^**^
Married	87.5	83.3	0.683^**^
**Educational level**			0.079^**^
Up to high school	29.2	54.2	
University degrees	70.8	45.8	
**Occupation**			0.456^**^
Student	4.1	12.5	
Housewife	41.7	33.3	
Employee	33.3	20.9	
Other	20.9	33.3	
**BMI status**			0.686^**^
< 35 Kg/m^2^	62.5	62.5	
35–39.9 Kg/m^2^	37.5	37.5	
**NAFLD severity**			0.558^**^
Mild	45.8	37.5	
Moderate	54.2	62.5	

MI, myo-inositol; NC, neck circumference; WC, waist circumference; BMI, body mass index; PAL, physical activity level; MET, total metabolic equivalents; NAFLD, non-alcoholic fatty liver disease. Mean (SD) is presented for data. ^*^p-value for independent sample *t*-test; ^**^p-value for the chi-square test.

Changes in dietary intakes, anthropometric measures, and physical activity levels over the study in the placebo and MI groups are presented in Table 2. Apart from DBP, no significant differences in anthropometric measures, physical activity, dietary intakes, and SBP were found between the groups at baseline. Anthropometric measures decreased significantly in both groups, while the reductions in weight (−4.72 Kg vs. −3.27 Kg, *p* = 0.049) and SBP (−4.37 mmHg vs. 0.83 mmHg, *p* = 0.006) in the MI group were significantly greater than the placebo group, after adjusting for potential confounders including baseline values and energy intake. Intra- and inter-group differences in physical activity were not statistically significant (Table 2). Although energy and macronutrient intakes decreased significantly in both groups, between-group differences were not significant after adjusting for the confounders. Therefore, physical activity and dietary macronutrient intakes were not considered confounders in data analysis.

**Table 2:** Anthropometric, blood pressure, physical activity, and dietary intakes of the study participants throughout the study.

	**Placebo (*n =* 24)**	**MI (*n =* 24)**	***P***
	**Mean** ±**SD**	**Mean** ±**SD**	
**Weight (Kg)**			
Baseline	96.93 ± 14.78	96.43 ± 14.68	0.907^**^
End	93.66 ± 15.16	91.71 ± 14.75	0.049^***^
MD (95 % CI)	3.27(1.99, 4.56)	4.72 (3.25, 6.19)	
*P* ^*^	**< 0.001**	**< 0.001**	
**BMI (Kg/m2)**			
Baseline	34.16 ± 3.38	33.77 ± 3.53	0.701
End	33.0 ± 3.46	32.11 ± 3.55	0.052
MD (95% CI)	1.16 (0.72, 1.60)	1.66 (1.16, 2.17)	
*P*	**< 0.001**	**< 0.001**	
**NC (cm)**			
Baseline	40.15 ± 3.76	39.54 ± 3.50	0.565
End	39.17 ± 3.62	38.06 ± 3.18	0.073
MD (95% CI)	0.98 (0.66, 1.30)	1.47 (1.00, 1.95)	
*P*	**< 0.001**	**< 0.001**	
**NC/height ratio**			
Baseline	0.24 ± 0.02	0.23 ± 0.02	0.371
End	0.23 ± 0.02	0.22 ± 0.01	0.061
MD (95% CI)	0.006 (0.004, 0.008)	0.009 (0.006, 0.011)	
*P*	< 0.001	< 0.001	
**NC/WC ratio**			
Baseline	0.36 ± 0.04	0.36 ± 0.03	0.903
End	0.37 ± 0.04	0.36 ± 0.03	0.329
MD (95% CI)	−0.01 (−0.01, 0.00)	−0.00 (−0.007, 0.005)	
*P*	0.081	0.777	
**SBP (mmHg)**			
Baseline	108.33 ± 11.67	114.17 ± 16.20	0.16
End	109.17 ± 11.00	109.79 ± 13.95	0.006
MD (95% CI)	−0.83 (−7.67, 6.00)	4.37 (−0.73, 9.48)	
*P*	0.803	0.09	
**DBP (mmHg)**			
Baseline	72.50 ± 11.98	81.04 ± 13.27	0.024
End	74.17 ± 11.00	72.29 ± 11.23	0.06
MD (95% CI)	−1.67 (−7.12, 3.78)	8.75 (3.35, 14.15)	
*P*	0.533	**0.003**	
**PAL (METs)**			
Baseline	57.54 ± 39.42	63.53 ± 37.17	0.59
End	54.79 ± 31.95	57.69 ± 30.70	0.819
MD (95% CI)	2.74 (−9.79, 15.27)	5.84 (−10.39,22.07)	
*P*	0.655	0.464	
**Energy (Kcal)**			
Baseline	2133.71 ± 797.53	2284.29 ± 866.15	0.534
End	1445.06 ± 421.60	1705.13 ± 827.83	0.177
MD (95% CI)	688.65 (448.61, 928.68)	579.17 (291.48, 866.85)	
*P* ^*^	**< 0.001**	**< 0.001**	
**Carbohydrates (g)**			
Baseline	309.24 ± 147.17	334.21 ± 154.41	0.57
End	216.68 ± 55.07	256.39 ± 178.45	0.307
MD (95% CI)	92.57 (40.62, 144.51)	77.83 (22.56, 133.10)	
*P*	**0.001**	**0.008**	
**Protein (g)**			
Baseline	80.97 ± 27.67	89.21 ± 34.95	0.37
End	60.00 ± 21.80	74.57 ± 39.60	0.123
MD (95% CI)	20.97 (11.08, 30.86)	14.64 (1.04, 28.24)	
*P*	**< 0.001**	**0.036**	
**Fat (g)**			
Baseline	69.63 ± 34.01	71.59 ± 32.46	0.839
End	41.51 ± 18.99	52.57 ± 39.81	0.226
MD (95% CI)	28.12 (16.46, 39.77)	19.02 (0.13, 37.92)	
*P*	**< 0.001**	**0.049**	
**Cholesterol (mg)**			
Baseline	240.91 ± 108.03	270.30 ± 172.73	0.483
End	194.48 ± 146.14	233.04 ± 239.33	0.504
MD (95% CI)	46.43 (−20.22, 113.08)	37.26 (−18.44, 92.96)	
*P*	0.163	0.18	
**SFA (g)**			
Baseline	21.18 ± 12.16	22.12 ± 11.40	0.783
End	11.27 ± 5.28	12.38 ± 6.50	0.518
MD (95% CI)	9.92 (5.35, 14.48)	9.74 (4.54, 14.95)	
*P*	**< 0.001**	**0.001**	
**MUFA(g)**			
Baseline	18.23 ± 9.59	20.54 ± 11.21	0.447
End	10.96 ± 5.26	12.79 ± 8.0845	0.355
MD (95% CI)	7.28 (3.62, 10.93)	7.75 (2.76, 12.74)	
*P*	**< 0.001**	**0.004**	
**PUFA (g)**			
Baseline	18.14 ± 13.23	16.72 ± 12.07	0.699
End	11.18 ± 10.23	15.86 ± 21.40	0.339
MD (95% CI)	6.96 (1.61, 12.30)	0.86 (−8.45, 10.18)	
*P*	**0.013**	0.85	
**Simple sugar (g)**			
Baseline	56.09 ± 29.67	61.67 ± 23.38	0.473
End	44.55 ± 33.25	47.50 ± 33.41	0.761
MD (95% CI)	11.54 (2.43, 20.63)	14.17 (4.19, 24.16)	
*P*	**0.015**	**0.007**	

MI, myo-inositol; BMI, body mass index; NC, neck circumference; WC, waist circumference; SBP, systolic blood pressure; DBP, diastolic blood pressure; PAL, physical activity level; METs, metabolic equivalents (MET-minutes/week); SFA, saturated fatty acid; MUFA, monounsaturated fatty acid; PUFA, polyunsaturated fatty acid. Mean (SD) and mean difference (95 % CI) are presented for data. ^*^p-value for paired *t*-test; ^**^p-value for independent samples *t*-test; ^***^p-value for ANCOVA test (adjusted for baseline values and energy intake). Bold values indicates statistically significant *p* < 0.05.

Table 3 shows changes in IR, lipid profile, and liver function parameters over the trial. Although the mean of HOMA-B was greater in the MI group compared to the placebo group at baseline (*p* = 0.027), other glucose-related factors did not show any significant differences. MI supplementation significantly decreased serum fasting glucose, insulin, HbA1c, and IR, while no changes were found in glucose homeostasis in the placebo group. After adjusting for baseline values and energy intake, inter-group differences in insulin and HOMA-IR reached a statistically significant level (*p* = 0.008 and 0.046, respectively).

**Table 3:** Cardiometabolic indices and liver parameters of the study participants throughout the study.

	**Placebo (*n =* 24)**	**MI (*n =* 24)**	***P***
	**Mean** ±**SD**	**Mean** ±**SD**	
**FBS (mg/dL)**			
Baseline	97.52 ± 12.26	91.59 ± 8.58	0.059^**^
End	96.31 ± 8.04	86.73 ± 6.28	0.263^***^
MD (95% CI)	1.21 (−2.68, 5.10)	4.87 (1.28, 8.45)	
*P* ^*^	0.525	**0.01**	
**Insulin (**μ**IU/mL)**			
Baseline	13.99 ± 7.77	16.62 ± 7.79	0.248
End	13.13 ± 4.56	12.50 ± 4.64	0.008
MD (95% CI)	0.86 (−3.08, 4.81)	4.12 (1.06, 7.19)	
*P* ^*^	0.655	**0.011**	
**HbA1c (%)**			
Baseline	5.41 ± 0.40	5.32 ± 0.46	0.465
End	5.37 ± 0.46	4.99 ± 0.44	0.186
MD (95% CI)	0.04 (−0.10, 0.17)	0.33 (0.15, 0.50)	
*P* ^*^	0.585	**0.001**	
**HOMA–IR**			
Baseline	3.42 ±2.07	3.81 ± 2.00	0.503
End	3.12 ±1.08	2.70 ± 1.08	0.046
MD (95% CI)	0.29 (−0.70, 1.28)	1.11 (0.33, 1.90)	
*P* ^*^	0.546	**0.007**	
**HOMA–B**			
Baseline	157.79 ± 80.47	219.45 ± 105.32	0.027
End	149.77 ± 68.04	194.12 ± 69.08	0.077
MD (95% CI)	8.02 (−36.05, 52.09)	25.34 (−20.08, 70.75)	
*P* ^*^	0.71	0.26	
**TC (mg/dL)**			
Baseline	186.13 ± 27.19	196.63 ± 40.87	0.301
End	175.21 ± 27.12	178.38 ± 36.77	0.01
MD (95% CI)	10.92 (2.68, 19.15)	18.25 (4.67, 31.83)	
*P* ^*^	**0.012**	**0.011**	
**TG (mg/dL)**			
Baseline	157.67 ± 57.85	181.40 ± 90.65	0.285
End	140.63 ± 50.59	169.32 ± 87.36	0.002
MD (95% CI)	17.04 (−3.29, 37.37)	12.07 (−17.98, 42.13)	
*P* ^*^	0.096	0.414	
**HDL-c (mg/dL)**			
Baseline	43.33 ± 9.39	43.82 ± 10.07	0.863
End	42.79 ± 6.61	45.60 ± 10.27	0.016
MD (95% CI)	0.54 (−3.62, 4.70)	−1.78 (−0.54, 1.80)	
P^*^	0.792	0.314	
**LDL-c (mg/dL)**			
Baseline	197.92 ± 29.79	204.16 ± 43.63	0.566
End	189.87 ± 30.08	190.11 ± 34.83	0.004
MD (95% CI)	8.04 (−0.54, 16.63)	14.06 (−1.75, 29.86)	
P^*^	0.065	0.079	
**AST (IU/L)**			
Baseline	28.50 ± 13.05	31.41 ± 16.53	0.501
End	21.38 ± 6.49	19.69 ± 4.30	0.066
MD (95% CI)	7.12 (2.44, 11.81)	11.72 (5.54, 17.90)	
*P* ^*^	**0.005**	**0.001**	
**ALT (IU/L)**			
Baseline	42.83 ± 30.99	37.42 ±23.36	0.498
End	28.56 ± 13.19	25.21 ± 10.10	0.027
MD (95% CI)	14.27 (4.14, 24.40)	12.21 (3.99, 20.44)	
*P* ^*^	**0.008**	**0.005**	
**AST/ALT**			
Baseline	0.77 ± 0.24	0.99 ± 0.40	0.024
End	0.81 ± 0.24	0.87 ± 0.28	0.006
MD (95% CI)	−0.05 (−0.14, 0.04)	0.12 (−0.01, 0.25)	
*P* ^*^	0.259	0.069	
**Ferritin (ng/mL)**			
Baseline	94.51 ± 84.42	93.61 ± 72.98	0.969
End	103.79 ± 91.58	77.49 ± 54.46	0.042
MD (95% CI)	−9.29 (−42.75, 24.17)	16.12 (−1.11, 33.36)	
*P* ^*^	0.571	0.065	

MI, myo-inositol; FBS, fasting blood sugar; HbA1c, glycosylated hemoglobin A1c; HOMA-IR, homeostatic model assessment of insulin resistance; HOMA-B, homeostasis beta-cell function; TC, total cholesterol; TG, triglyceride; HDL-C, high-density lipoprotein cholesterol; LDL-C, low-density lipoprotein cholesterol; AST, aspartate aminotransferase; ALT, alanine transaminase; AST/ALT, AST-to-ALT ratio. Mean (SD) and mean difference (95 % CI) are presented for data. ^*^p-value for paired *t*-test; ^**^p-value for independent samples *t*-test; ^***^p-value for ANCOVA test (adjusted for baseline values and energy intake). Bold values indicates statistically significant *p* < 0.05.

Indeed, after adjusting for baseline values and energy intake, MI supplementation resulted in improvements in all lipid factors [TC (*p* = 0.010), TG (*p* = 0.002), LDL-c (*p* = 0.004), and HDL-c (*p* = 0.016)]. Moreover, supplementation with MI reduced serum ALT (*p* = 0.027), AST (*p* = 0.066), and AST/ALT ratio (*p* = 0.006). The serum ferritin level noticeably decreased in the MI group (*p* = 0.042) compared to the placebo at the endpoint, considering the role of the potential confounders (Table 3).

The clinical effectiveness of MI supplementation on liver steatosis severity has been shown in Table 4. MI conferred a 33.3 and 12.5% ARR in the reduction of steatosis severity. The estimated NNT for 1-grade and 2-grade reduction in steatosis severity was 3 and 8, i.e., of every three and eight patients with NAFLD who supplemented with MI (4g/day) for 8 weeks, one patient would experience one-grade reduction in liver steatosis and treated completely (2-grade reduction), respectively.

**Table 4:** Effectiveness level for reduction in steatosis severity.

**Reduction in NAFLD severity**	**Group**	***N* (%)**	**ARR (%)**	**NNT**	*P* ^*^
1 grade	MI	12 (50.0)	33.3	3	0.014
	Placebo	4 (16.7)			
2 grades	MI	3 (12.5)	12.5	8	0.234
	Placebo	0 (0)			

MI, myo-inositol; ARR, absolute risk reduction; NNT, number needed to treat. ^*^p-value for the chi-square test.

## Discussion

The results of the present RCT on the effect of MI supplementation in NAFLD for 8 weeks showed considerable improvements in IR, lipid profile, and liver steatosis. MI supplementation accompanied by dietary recommendations resulted in a significant reduction in dietary intakes of energy and macronutrients and an average weight loss of 4.72 Kg in the MI group vs. 3.27 Kg in the placebo group (Table 2). Although no significant differences in physical activity and reduction in energy intake (−579 Kcal and −688 Kcal in the MI group and placebo group, respectively) were found between the groups at the end of the study, the greater reductions in weight, BMI, NC, and NC/height ratio in the MI group are attributed to MI supplementation, after adjusting for the confounders. There is evidence illustrating that INS derivatives, particularly inositol hexakisphosphate (IP6) could decrease weight through increasing serum leptin which in turn, could regulate food intake in T2DM rats (27). INS phosphates reduced fat deposits and body weight *via* increased oxygen consumption and energy expenditure but without any changes in energy intake in IP6 kinase knockout mice (28). NC—a simple inexpensive, non-invasive, and quick assessment, can reflect subcutaneous fat accumulation and obesity, particularly mid-upper body obesity (29). NC is also linked to metabolic disorders such as CVD and atherosclerosis as well as influenced by changes in insulin levels (29). A recent meta-analysis reported not only the beneficial effects of INSs on BMI reduction but also a stronger association of MI with BMI reduction compared to other derivatives of INS (30). Moreover, SBP was significantly reduced in the MI group compared to the placebo group (*p* = 0.006), while no significant inter-group difference was observed in DBP (Table 2). Nestler et al. (31) reported that D-Chiro-INS (DCI) administration improves not only IR and plasma TG levels but also blood pressure in women with PCOs. Tari et al. (32) in their meta-analysis on seven RCTs reported the potential beneficial effects of supplementation with MI, DCI, and pinitol (0.6–4 g/day) on blood pressure, particularly in those with Mets for a longer duration. The proposed mechanism for the hypotensive property of INSs is related to releasing nitric oxide and a subsequent vasodilator effect as well as downregulating nuclear factor-kB gene expression, reducing pro-inflammatory cytokines and thereby lowering blood pressure (32).

There is cumulative evidence regarding the favorable effect of supplementation with INS derivatives on IR, particularly in T2DM, PCOs, and gestational diabetes mellitus (GDM). These findings could be attributed to patients with NAFLD due to the significant link between T2DM and NAFLD, through their mutual risk factors (5). Our results demonstrated a significant reduction in serum insulin levels and HOMA-IR as well as a decrease but not statistically significant in HOMA-B after adjusting for potential confounders (Table 3) which are in agreement with previous animal (33) and human studies (34). A systematic review and meta-analysis on 20 RCTs including 1,239 subjects with different types of IR revealed that INS supplementation resulted in decreased FBS, insulin, and HOMA-IR (35). Swathi et al. (36) showed that MI supplementation plus diet was more effective in glucose reduction than diet alone in patients with GDM. MI treatment has been shown to improve IR (assessed by HOMA-IR), metabolic, and hormonal disturbances in PCOs (37). A meta-analysis on nine RCTs also reported a significant reduction in IR after 12–24 week supplementation with MI in patients with PCOs (38). Moreover, MI supplementation compared with metformin and oral contraceptive revealed similar or even greater effectiveness with fewer side effects in improving IR (39). The underlying mechanism of insulin-sensitizing effect of MI could be explained by (1) activating PI3K/AKT signaling pathway, followed by glycogen synthase stimulation, (2) increasing glucose transporter type 4 (GLUT-4) translocation to the cell membrane and thereby improving glucose uptake, (3) enhancing the insulin receptor substrate activation, (4) amelioration of the adverse effects of chronic insulin stimulation in adipocytes, (5) insulin-mimetic property, and (6) reducing glucose absorbance and post-prandial glucose circulation and reducing intestinal transit time (14) in an independent manner from weight reduction (34). Our results also showed beneficial effects of MI on lipid profile (Table 3), i.e., significant reductions in serum levels of TG, TC, and LDL-c as well as a marked increase in serum HDL-c. Animal studies have shown lowering TG accumulation in the liver and serum lipids of different types of INSs (mostly MI and pinitol) (19). Furthermore, pinitol supplementation (300 and 500 mg/day) in subjects with NAFLD for 12 weeks resulted in a slight but not significant reduction in TC and LDL-c levels, andthe lowest level of TG was found in high dose pinitol (20). The proposed mechanism of pinitols could be explained by alleviating oxidative stress and fatty acid deposition, leading to the regulation of energy and lipid metabolisms (20) which, in turn, positively improves liver dysfunction by reducing the oxidative stress in hepatic tissues (40). A systematic review of 14 RCTs on patients with metabolic conditions reported improvements in lipid profile after MI supplementation without any significant effect on HDL-c level (17). INSs seem to improve lipid oxidation and profile by improving AMP-activated protein kinase (AMPK) activity (33).

The present results also found the positive effects of MI supplementation on liver function in terms of serum liver enzymes, AST/ALT ratio, and ferritin (Table 3). Genetic and epigenetic factors simultaneously affect IR (as the focal hit of NAFLD) and hepatic enzymes and, therefore, lead to liver dysfunction, characterized by increased ALT, AST, gamma-glutamyl transferase (GGT), bilirubin, and ferritin in NAFLD (5, 41). Zhou et al. (42) showed that pinitol supplementation suppressed increased ALT and AST in rats fed by high- fat diet with hepatic injury. Indeed, Lee et al. (20) reported a reduction in liver steatosis and enzymes after 300 and 500 mg/day pinitol supplementation for 12 weeks in NAFLD. Liver iron is an important source of serum ferritin and is considered the main prognostic marker for NAFLD (43). Moreover, serum ferritin is closely related to IR, inflammation, NAFLD incidence, and the severity of fat accumulation in the liver and increased liver enzymes (44). INS in the form of IP6 is considered an iron chelator in hyperferritinemia and thalassemia (45). There is evidence indicating hyperferritinemia with normal transferrin saturation is an indicator of glucose–lipid metabolism disorders (46).

From a clinical point of view, as specialists are interested in therapy options focusing on the benefit to risks, our results found that the estimated NNTs (Table 4) for one- and two-grade reductions in liver steatosis also present an improvement in liver function.

To the best of our knowledge, the current trial appears to be the first human study aimed to investigate the effectiveness of MI supplementation along with dietary recommendations on cardiometabolic factors and liver function in obese patients with NAFLD. Considering possible confounders, applying dietary recommendations for weight loss as a confirmed approach in NAFLD management, using a placebo as well as frequent visits, and following up by phone calls for increasing compliance are considered the major strengths of the trial. However, the relatively short study duration, the conservative MI dose selected, and studying only patients with obesity as well as subjectively assessing physical activity and dietary intake could be counted as the limitations of the present study.

## Conclusion

It is concluded that MI supplementation at a dose of 4 g/day for 8 weeks significantly improves not only IR and lipid profile but also liver function in NAFLD. Because of the limited RCTs in humans, further clinical trials with larger sample sizes, longer duration, different MI doses, and other inositol derivatives are recommended.

## Data availability statement

The original contributions presented in the study are included in the article/supplementary material, further inquiries can be directed to the corresponding author.

## Ethics statement

The studies involving human participants were reviewed and approved by the Research Vice-Chancellor of Tabriz University of Medical Sciences. The patients/participants provided their written informed consent to participate in this study.

## Author contributions

SA and SR help in data collection. MK contributed to patient selection. SA wrote the original manuscript. SA and ME-M carried out the statistical analysis. ME-M, NR, and HT contributed to the conception of the article and the final revision of the manuscript. All authors read and approved the final version of the manuscript.

## References

[1] Ramai D Tai W Rivera M Facciorusso A Tartaglia N Pacilli M. Natural progression of non-alcoholic steatohepatitis to hepatocellular carcinoma. Biomedicines. (2021) 9:184. 10.3390/biomedicines9020184 33673113 PMC7918599

[2] Estes C Razavi H Loomba R Younossi Z Sanyal AJ. Modeling the epidemic of nonalcoholic fatty liver disease demonstrates an exponential increase in burden of disease. Hepatology. (2018) 67:123–33. 10.1002/hep.29466 28802062 PMC5767767

[3] Chalasani N Younossi Z Lavine JE Diehl AM Brunt EM Cusi K. The diagnosis and management of non-alcoholic fatty liver disease: Practice Guideline by the American Association for the Study of Liver Diseases, American College of Gastroenterology, and the American Gastroenterological Association. Hepatology. (2012) 55:2005–23. 10.1002/hep.25762 22488764

[4] Marchisello S Di Pino A Scicali R Urbano F Piro S Purrello F. Pathophysiological, molecular and therapeutic issues of nonalcoholic fatty liver disease: An overview. Int J Mol Sci. (2019) 20:1948. 10.3390/ijms20081948 31010049 PMC6514656

[5] Sung K-C Jeong W-S Wild SH Byrne CD. Combined influence of insulin resistance, overweight/obesity, and fatty liver as risk factors for type 2 diabetes. Diabetes Care. (2012) 35:717–22. 10.2337/dc11-1853 22338098 PMC3308286

[6] Bazick J Donithan M Neuschwander-Tetri BA Kleiner D Brunt EM Wilson L. Clinical model for NASH and advanced fibrosis in adult patients with diabetes and NAFLD: guidelines for referral in NAFLD. Diabetes Care. (2015) 38:1347–55. 10.2337/dc14-1239 25887357 PMC4477334

[7] Marušić M Paić M Knobloch M Liberati Pršo A-M. NAFLD, insulin resistance, and diabetes mellitus type 2. Canad J Gastroenterol Hepatol. (2021) 2021:6613827. 10.1155/2021/6613827 33681089 PMC7904371

[8] Koliaki C Szendroedi J Kaul K Jelenik T Nowotny P Jankowiak F. Adaptation of hepatic mitochondrial function in humans with non-alcoholic fatty liver is lost in steatohepatitis. Cell Metab. (2015) 21:739–46. 10.1016/j.cmet.2015.04.004 25955209

[9] Younossi Z Tacke F Arrese M Chander Sharma B Mostafa I Bugianesi E. Global perspectives on nonalcoholic fatty liver disease and nonalcoholic steatohepatitis. Hepatology. (2019) 69:2672–82. 10.1002/hep.30251 30179269

[10] Tarantino G Savastano S Colao A. Hepatic steatosis, low-grade chronic inflammation and hormone/growth factor/adipokine imbalance. World J Gastroenterol. (2010) 16:4773. 10.3748/wjg.v16.i38.4773 20939105 PMC2955246

[11] Younossi ZM Rinella ME Sanyal AJ Harrison SA Brunt EM Goodman Z. From NAFLD to MAFLD: implications of a premature change in terminology. Hepatology. (2021) 73:1194–8. 10.1002/hep.31420 32544255

[12] Friedman SL Neuschwander-Tetri BA Rinella M Sanyal AJ. Mechanisms of NAFLD development and therapeutic strategies. Nat Med. (2018) 24:908–22. 10.1038/s41591-018-0104-9 29967350 PMC6553468

[13] DiNicolantonio JJ O'Keefe JH. Myo-inositol for insulin resistance, metabolic syndrome, polycystic ovary syndrome and gestational diabetes. Arch Dis Childh. (2022) 9:e001989. 10.1136/openhrt-2022-001989 35236761 PMC8896029

[14] Bevilacqua A Bizzarri M. Inositols in insulin signaling and glucose metabolism. Int J endocrinol. (2018) 2018:1968450. 10.1155/2018/1968450 30595691 PMC6286734

[15] Clements Jr RS Darnell B. Myo-inositol content of common foods: development of a high-myo-inositol diet. Am J Clin Nutr. (1980) 33:1954–67. 10.1093/ajcn/33.9.1954 7416064

[16] Chatree S Thongmaen N Tantivejkul K Sitticharoon C Vucenik I. Role of inositols and inositol phosphates in energy metabolism. Molecules. (2020) 25:5079. 10.3390/molecules25215079 33139672 PMC7663797

[17] Tabrizi R Ostadmohammadi V Lankarani KB Peymani P Akbari M Kolahdooz F. The effects of inositol supplementation on lipid profiles among patients with metabolic diseases: a systematic review and meta-analysis of randomized controlled trials. Lipids Health Dis. (2018) 17:1–11. 10.1186/s12944-018-0779-4 29793496 PMC5968598

[18] Croze ML Soulage CO. Potential role and therapeutic interests of myo-inositol in metabolic diseases. Biochimie. (2013) 95:1811–27. 10.1016/j.biochi.2013.05.011 23764390

[19] Pani A Giossi R Menichelli D Fittipaldo VA Agnelli F Inglese E. Inositol and non-alcoholic fatty liver disease: A systematic review on deficiencies and supplementation. Nutrients. (2020) 12:3379. 10.3390/nu12113379 33153126 PMC7694137

[20] Lee E Lim Y Kwon SW Kwon O. Pinitol consumption improves liver health status by reducing oxidative stress and fatty acid accumulation in subjects with non-alcoholic fatty liver disease: A randomized, double-blind, placebo-controlled trial. J Nutr Biochem. (2019) 68:33–41. 10.1016/j.jnutbio.2019.03.006 31030165

[21] Hamaguchi M Kojima T Itoh Y Harano Y Fujii K Nakajima T. The severity of ultrasonographic findings in nonalcoholic fatty liver disease reflects the metabolic syndrome and visceral fat accumulation. ACG. (2007) 102:2708–15. 10.1111/j.1572-0241.2007.01526.x 17894848

[22] Evert AB Boucher JL Cypress M Dunbar SA Franz MJ Mayer-Davis EJ. Nutrition therapy recommendations for the management of adults with diabetes. Diabetes Care. (2014) 37:S120–S43. 10.2337/dc14-S120 24357208

[23] Committee IR,. Guidelines for Data Processing Analysis of the International Physical Activity Questionnaire (IPAQ)-Short Long Forms (2005). Available online at: http://www.ipaq.ki.se/scoring.pdf (accessed September 17, 2008).

[24] Friedewald WT Levy RI Fredrickson DS. Estimation of the concentration of low-density lipoprotein cholesterol in plasma, without use of the preparative ultracentrifuge. Clin Chem. (1972) 18:499–502. 10.1093/clinchem/18.6.499 4337382

[25] Schumann G Klauke R Canalias F Bossert-Reuther S Franck PF Gella F-J. IFCC primary reference procedures for the measurement of catalytic activity concentrations of enzymes at 37 C. Part 9: Reference procedure for the measurement of catalytic concentration of alkaline phosphatase. Clin Chem Lab Med. (2011) 49:1439–46. 10.1515/CCLM.2011.621 21702699

[26] Matthews DR Hosker J Rudenski A Naylor B Treacher D Turner R. Homeostasis model assessment: insulin resistance and β-cell function from fasting plasma glucose and insulin concentrations in man. Diabetologia. (1985) 28:412–9. 10.1007/BF00280883 3899825

[27] Foster SR Omoruyi FO Bustamante J Lindo RL Dilworth LL. The effect of combined inositol hexakisphosphate and inositol supplement in streptozotocin-induced type 2 diabetic rats. Int J Exp Pathol. (2016) 97:397–407. 10.1111/iep.12210 27921351 PMC5206813

[28] Chakraborty A Koldobskiy MA Bello NT Maxwell M Potter JJ Juluri KR. Inositol pyrophosphates inhibit Akt signaling, thereby regulating insulin sensitivity and weight gain. Cell. (2010) 143:897–910. 10.1016/j.cell.2010.11.032 21145457 PMC3052691

[29] Jian C Xu Y Ma X Shen Y Wang Y Bao Y. Neck circumference is an effective supplement for nonalcoholic fatty liver disease screening in a community-based population. Int J Endocrinol. (2020) 2020:7982107. 10.1155/2020/7982107 32508918 PMC7246413

[30] Zarezadeh M Dehghani A Faghfouri AH Radkhah N Naemi Kermanshahi M Hamedi Kalajahi F. Inositol supplementation and body mass index: A systematic review and meta-analysis of randomized clinical trials. Obesity Sci Pract. (2022) 8:387–97. 10.1002/osp4.569 35664247 PMC9159559

[31] Nestler JE Jakubowicz DJ Reamer P Gunn RD Allan G. Ovulatory and metabolic effects of D-chiro-inositol in the polycystic ovary syndrome. New England J Med. (1999) 340:1314–20. 10.1056/NEJM199904293401703 10219066

[32] Tari SH Sohouli MH Lari A Fatahi S Rahideh ST. The effect of inositol supplementation on blood pressure: A systematic review and meta-analysis of randomized-controlled trials. Clin Nutr ESPEN. (2021) 44:78–84. 10.1016/j.clnesp.2021.06.017 34330516

[33] Antony PJ Gandhi GR Stalin A Balakrishna K Toppo E Sivasankaran K. Myoinositol ameliorates high-fat diet and streptozotocin-induced diabetes in rats through promoting insulin receptor signaling. Biomed Pharmacother. (2017) 88:1098–113. 10.1016/j.biopha.2017.01.170 28192884

[34] Kim J-I Kim J Kang M-J Lee M-S Kim J-J Cha I-J. Effects of pinitol isolated from soybeans on glycaemic control and cardiovascular risk factors in Korean patients with type II diabetes mellitus: a randomized controlled study. Eur J Clin Nutr. (2005) 59:456–8. 10.1038/sj.ejcn.1602081 15536472

[35] Miñambres I Cuixart G Gonçalves A Corcoy R. Effects of inositol on glucose homeostasis: systematic review and meta-analysis of randomized controlled trials. Clin Nutr. (2019) 38:1146–52. 10.1016/j.clnu.2018.06.957 29980312

[36] Swathi B Deepthi A Sravani B Namratha S Sandhya R A. prospective comparative study to evaluate the effect of Myo-inositol plus diet vs diet alone in patients with gestational diabetes mellitus. GSC Biol Pharmac Sci. (2021) 14:197–201. 10.30574/gscbps.2021.14.3.0076

[37] Kamenov Z Gateva A. Inositols in PCOS. Molecules. (2020) 25:5566. 10.3390/molecules25235566 33260918 PMC7729761

[38] Unfer V Facchinetti F Orrù B Giordani B Nestler J. Myo-inositol effects in women with PCOS: a meta-analysis of randomized controlled trials. Endocrine Connect. (2017) 6:647–58. 10.1530/EC-17-0243 29042448 PMC5655679

[39] Facchinetti F Orru B Grandi G Unfer V. Short-term effects of metformin and myo-inositol in women with polycystic ovarian syndrome (PCOS): a meta-analysis of randomized clinical trials. Gynecol Endocrinol. (2019) 35:198–206. 10.1080/09513590.2018.1540578 30614282

[40] Barman S Srinivasan K. Attenuation of oxidative stress and cardioprotective effects of zinc supplementation in experimental diabetic rats. Br J Nutr. (2017) 117:335–50. 10.1017/S0007114517000174 28245884

[41] De Silva NMG Borges MC Hingorani AD Engmann J Shah T Zhang X. Liver function and risk of type 2 diabetes: bidirectional mendelian randomization study. Diabetes. (2019) 68:1681–91. 10.2337/db18-1048 31088856 PMC7011195

[42] Zhou Y Park C-M Cho C-W Song Y-S. Protective effect of pinitol against D-galactosamine-induced hepatotoxicity in rats fed on a high-fat diet. Biosci Biotechnol Biochem. (2008) 72:1657–66. 10.1271/bbb.70473 18603811

[43] Bhowmik A Ojha D Goswami D Das R Chandra NS Chatterjee TK. Inositol hexa phosphoric acid (phytic acid), a nutraceuticals, attenuates iron-induced oxidative stress and alleviates liver injury in iron overloaded mice. Biomed Pharmacother. (2017) 87:443–50. 10.1016/j.biopha.2016.12.125 28068635

[44] Yan J-X Pan B-J Zhao P-P Wang L-T Liu J-F Fu S-B. Serum ferritin is correlated with non-alcoholic fatty liver disease in middle-aged and older patients with type 2 diabetes. Endocrine Connect. (2021) 10:1560–9. 10.1530/EC-21-0367 34738917 PMC8679874

[45] Ryan JD Armitage AE Cobbold JF Banerjee R Borsani O Dongiovanni P. Hepatic iron is the major determinant of serum ferritin in NAFLD patients. Liver Int. (2018) 38:164–73. 10.1111/liv.13513 28679028

[46] Lombardi R Pisano G Fargion S. Role of serum uric acid and ferritin in the development and progression of NAFLD. Int J Mol Sci. (2016) 17:548. 10.3390/ijms17040548 27077854 PMC4849004

